# Migratory routes, breeding locations and multiple non-breeding sites of Common Whitethroats *Curruca communis* revealed by geolocators

**DOI:** 10.1371/journal.pone.0274017

**Published:** 2022-09-01

**Authors:** Claudia Tapia-Harris, Arin Izang, Will Cresswell

**Affiliations:** 1 Centre for Biological Diversity, School of Biology, University of St Andrews, St Andrews, United Kindom; 2 A.P. Leventis Ornithological Research Institute, Jos, Nigeria; Liverpool John Moores University, UNITED KINGDOM

## Abstract

Understanding general migration characteristics and how breeding and non-breeding sites are connected is crucial for predicting the response of long-distance migratory bird populations to environmental changes. We use data collected from six geolocators to describe migratory routes and identify breeding and non-breeding locations, migratory behaviour and differences between spring and autumn migration of Common Whitethroats *Curruca communis*, an Afro-Palearctic migrant, wintering in Nigeria. Most individuals departed on spring migration in April, following a north-easterly direction, arriving at their breeding grounds across central-eastern Europe (~425,000 km^2^) in May. Departures from breeding grounds took place between July and August in a south-westerly direction. During spring migration individuals travelled longer distances at faster rates making its overall duration shorter than autumn migration. We suggest that, while Whitethroats can cross the Sahara Desert and Mediterranean Sea in a single flight, they are likely to refuel before and after crossing. Results indicate that Whitethroats undertook loop migration and visited two wintering sites: first in the Sahel, then in Nigeria, where they remained until spring migration. Geolocator results and data from the European Union for Bird Migration’s (EURING) ringing database suggest that Whitethroats have a relatively high migratory spread—individuals from a single non-breeding site breed across a wide area of Europe. Our research is the first to track and describe the complete annual cycle of Whitethroats and one of the few studies to do so for any Afro-Palearctic migrant from non-breeding grounds. We identified the Sahel as an important refuelling and first wintering site indicating its conservation, alongside other stopover sites, is crucial for the species. We believe that changes in this region will have severe effects on a subset of individuals of specific European breeding populations, but these effects will greatly depend on the severity of the changes and at what spatial scale they occur.

## Introduction

The survival of Afro-Palearctic migratory birds is influenced by conditions at breeding and non-breeding grounds as well as along migratory routes [1, 2]. Studying and understanding the full annual cycle of migrants is therefore crucial for recognising and mitigating threats and for identifying conservation priorities, especially in light of recent global population declines and increasing habitat and environmental changes that most migrants face throughout their annual cycle [3, 4].

Key to identifying where, when, and how populations of migrants are limited is in identifying how different breeding populations spread and mix during the non-breeding period (‘migratory spread’ or ‘migratory connectivity’), and in understanding the routes individuals undertake from breeding grounds to non-breeding grounds and back. For example, species with low migratory spread, where most individuals from a breeding population winter at the same location, are less likely to adapt to shifting habitats and can be less resilient to global changes than species with higher migratory spread, where individuals from same breeding population winter at several locations and mix with individuals from other breeding populations [5, 6].

The conditions and time spent en route additionally strongly influence an individual’s survival, particularly when crossing major ecological barriers (e.g. Sahara Desert and Mediterranean Sea), where migrants encounter harsh environmental conditions for > 2000 km, considering the Sahara exclusively [7–9]. To cope with these conditions, birds either accumulate enough body reserves to carry out a long, quick flight, migrating in a single journey (‘non-stop’) or break the journey into sections refuelling along the way (‘intermittent’) [10–12]. The former migration behaviour decreases migration time and exposure to threats but requires high body reserves before starting the journey and increases predation risk due to reduced mobility, while the latter increases exposure to a wider range of threats (e.g. competition and pathogen and parasite exchange), mainly at stopover sites, but fuel-use and recovery rates are more efficient. The quality of stopover sites when using intermittent migration will determine refuelling rates and duration spent at them, which in turn will determine the number of stopover sites utilised, arrival dates at breeding and non-breeding grounds, migration success, and overall survival [12, 13].

The composition and quality of stopover sites, however, varies seasonally and annually. In many African regions, habitats change considerably throughout and across years [14]. The difference in migratory behaviours therefore also reflects how species overcome seasonal changes and how flexible they can be regarding the number of stopovers used and the time spent at each site. Additionally, to cope with significant productivity changes throughout the year, and to take advantage of seasonal differences in wind pattern (i.e. speed and direction), many Afro-Palearctic migrants often follow different routes during spring and autumn (‘loop migration’) [15–17].

Understanding the implications of migratory spread and migration timing and routes for a given population requires detailed knowledge of where breeding birds go during the non-breeding season and vice versa. For many years, recaptures and sporadic resightings were the only source of knowledge regarding large-scale small bird movements and most bird movements in Africa. However, the development of smaller and cheaper tracking devices, such as light-level geolocators (hereafter ‘geolocators’), have increased bird migration knowledge, as individual birds can now be tracked throughout the entirety of their annual cycle. Geolocators, which are currently the only option for tracking long-distance movements of small passerine birds [18] record light intensity at regular time intervals. This allows for the inference of solar positions that, in turn, can be used to calculate latitude and longitude from the length of the solar day and from the time of local solar noon, respectively [18, 19]. Despite their disadvantages (e.g. need to recapture individuals to download data and low geographical position resolution due to local variation in sun elevation angles), geolocators reveal new information regarding migration routes and breeding and non-breeding locations of specific individuals, making them highly suitable for the study of the migration of small birds [20].

Although there has been an increase in the number of studies using geolocators, until now few migratory species have been tracked from the non-breeding grounds [21–23] and most have been deployed on populations in the Neotropics. By deploying geolocators at non-breeding sites on species that are territorial during this period, data regarding migratory spread can be collected more accurately because latitude estimation is more accurate towards the poles, and birds are more likely to be stationary during the breeding period [20]. Such studies also give insight into the breeding origins of birds sharing the same non-breeding sites [23].

The Common Whitethroat *Curruca communis* (henceforth ‘Whitethroat’) is a widely distributed Afro-Palearctic migrant that breeds from the Arctic Circle to Morocco, and from Ireland to central Siberia, and spends the non-breeding season in sub-Saharan Africa, from Senegal to Ethiopia and from Ethiopia to South Africa along the eastern coast [24, 25]. It has been suggested that individuals move from one stopover to the next, accumulating just the necessary fat to do so [26, 27], but can also fly across the Sahara Desert and the Mediterranean Sea without refuelling [28]. Data obtained through ring recoveries from 150 individuals, mostly during migration, indicate that more western breeding populations spend the non-breeding period in western Africa, while more eastern breeding populations winter in central and eastern Africa [29], but much information is still lacking regarding general migration details.

In this study, we use data collected from geolocators to describe migratory routes, locate breeding, wintering, and key stopover locations, and describe migratory behaviours and differences between spring and autumn migration of Whitethroats wintering in Nigeria. We provide the first description of the complete annual cycle of individuals of this species and are one of the few studies to do so for any Afro-Palearctic migrant at non-breeding grounds.

## Methods

### Study site, geolocator deployment and retrieval

This study was conducted within a six km^2^ area at a Guinea savannah at the A.P. Leventis Ornithological Research Institute (APLORI) on the Jos Plateau, central Nigeria in West Africa (09°52’N, 08°58’E, 1250 masl) during the region’s dry season. The landscape mainly consisted of open scrublands with scattered bushes and grasses and varying degrees of anthropogenic activities such as farming, livestock grazing, human settlements, tin mining and fires, intensities of which varied throughout the season [30]. The study area has high Whitethroat densities and represents typical African dynamic habitats where anthropogenic activities are constant and continuously changing throughout the year.

Between January and March 2019, 60 Whitethroats (see S1 Table for more geolocator details and photographs) were captured with mist-nets, marked with one metal and three colour rings for facilitating relocation, and deployed with geolocators. Forty individuals were fitted with ML6740 geolocators (~0.51g, 5 mm light-stalk positioned at a 45° fixed angle; developed by the British Antarctic Survey), ten with FL6B57 geolocators (~0.40 g, 5 mm light-stalk; developed by Lotek/Biotrack, UK) and ten with FL6057 geolocators (~0.37 g, no light-stalk; Lotek/Biotrack, UK). Geolocators were attached using an elastic leg-loop harness following [31]. On average, devices weighed 0.5 g (including harness), representing 3.7% of average body mass. To estimate the effects geolocators had on birds, 60 additional control individuals were captured, handled, and marked during the same period but were not deployed with geolocators.

Individuals were sought out between November 2019 and March 2020. Two observers undertook resightings at least once a week between sunrise and ~1030 hrs and/or between ~1500 hrs and sunset using 10 x 40 binoculars. When identified, individuals were captured, geolocators were removed by cutting the harness and birds were released unharmed. This work was conducted under the ethical guidelines of the A.P. Leventis Ornithological Research Institute Scientific Committee and all methods were approved by the School of Biology Ethics Committee of the University of St. Andrews (SEC17028). Seven geolocators were recovered (six ML6740 and one FL6B57); data were obtained from six ML6740 devices; five with complete annual-cycle information—three females, two males—and one with partial information corresponding to spring migration, a female whose battery ran out on 10 June 2019, after arriving at its breeding ground. No data could be obtained from the FL6B57 tag due to tag damage. All seven individuals were adults when geolocators were deployed.

Overall, there was no strong evidence for tag effects. Although return rates differed slightly between geolocated and control individuals, these differences were not statistically significant. Return rates were similar between all geolocated individuals and controls (geolocated *n* = 7/60, 11.7%; controls *n* = 9/60, 15%; χ^2^ = 0.3, df = 1, *p* = .60) and between geolocated and control adult birds (geolocated *n* = 7/35, 20%; controls *n* = 5/29, 17.2%; χ^2^ = 0.08, df = 1, *p* = .78). Although we did not detect any first-year geolocated bird the following year, there was no statistical difference between return rates of geolocated and control first-year individuals (geolocated *n* = 0/24, 0%; controls *n* = 4/29, 13.8%; Fisher’s Exact Test *p* = .12). Birds that carried geolocators model “ML” had higher return rates (*n* = 6/40, 15%) than those carrying “FL” geolocators (*n* = 1/20, 5%), but this difference was not statistically significant (Fisher’s Exact Test *p* = .41). The mean weight of recovered geolocated birds was similar to controls captured during the same period (geolocated = 14.05 g, controls = 14.4 g; two sample *t*-test: *t* = 0.8, df = 9.5, *p* = .43).

All individuals survived at least until their departure on spring migration once geolocators were retrieved. Overall, our evidence, although of limited statistical power, does not suggest that tagging had a strong negative effect on the survival of this species in this particular area and is consistent with results from other studies [32, 33] but the use of geolocators should always be treated with caution [34, 35].

### Geolocator analysis

Raw geolocator data were downloaded and linearly corrected for clock drift using the *BASTrak* software (British Antarctic Survey) following the BAS Geolocator Manual [36]. After decompressing the raw data, *TransEdit2* software was used to obtain daily sunrises and sunsets using a single light threshold value of two, which is close to civil twilight. We set the minimum dark period filter to 4 hours to eliminate evidently false twilights. Data were then visually inspected to ensure that two positions—one sunrise and sunset—were obtained each day. When positions seemed clearly different from previous/latter values and were therefore likely to be erroneous, new values were obtained by averaging the sunrise/sunset of the day before and after. Further analyses were carried out using R version 3.6.3 [37]. Sunrise and sunset data were further filtered with the *loessFilter* function from the *GeoLight* package (version 2.0.0), which validates twilights and identifies possible shading events caused by topography, weather, vegetation, and/or behaviour [38] and thus are considered erroneous (by identifying residuals that were greater than three times the interquartile range) [39]. We visually inspected results and manually eliminated false coordinates that were 20 min different from prior and latter values during stationary periods. These steps removed 0–7.4% of locations (mean = 2.5%).

We then used the *getElevation* function of *GeoLight* to obtain the sun-elevation angle (SEA) from the known non-breeding location for the period during which we were certain the bird remained at the site (‘on-bird in-habitat calibration’; mean 51 days from deployment to last seen, range 19–67 days), except for individuals 094 and 108, who were not seen after deployment, for whom we therefore used a two-week period after deployment for calibration. Because it is impossible to know the physical conditions outside of the non-breeding grounds, three SEAs were used to estimate latitudes in these locations: the exact SEA provided by the *getElevation* function and its closest upper and lower integer number, ranging between -2° and -5°; -4° represented the median and mean SEA of all five individuals). We used the individual SEA that best located our known non-breeding site (APLORI), which varied according to each individual (range between -3° and -4.2°, median and mean = -3.85°) and was used throughout their respective annual cycle. Breeding sites were, on average, 150 km apart when using all three possible SEA values.

Stationary and migratory periods were determined using the *changeLight* function of *GeoLight*, setting the minimal stopover period to three days and the quantile probability threshold to .95 [39]. We then used the *mergeSites* function to combine consecutive sites separated by 150 km, which are likely to be the same location, but which the function separates due to errors in twilight times. All twilight values were converted to geographic coordinates using the *coord* function. We then averaged the 4-day rolling average for each stationary site. Latitude is derived from day length while longitude is derived from the absolute time of local noon and midnight on a given date. Because latitude is difficult to estimate around the equinox periods, when the difference in day and night length is minimal, we excluded data for 14 days before and after the vernal and autumnal equinoxes (20 March and 23 September 2019, respectively) from further analyses. Given that birds were still at the wintering grounds during the spring equinox, this only affected results from autumn migration. Each individual’s data were run with different SEAs, and *mergeSites* settings (50, 100, 150, and 200 km). We believe that final parameter selection reasonably describes our species migratory routes and that varying these parameters does not greatly change the biological or statistical significance of the results. As very few devices were retrieved, due to low statistical power, we could not make any inferences regarding age and sex differences.

### EURING long-term ringing recoveries

To complement our geolocator data and to further understand migratory spread, in February 2021 we requested to the European Union for Bird Ringing (EURING) access to all records of Whitethroats that were detected at least once in both Europe and Africa or at least twice in Africa. We originally obtained information from 243 individuals. We eliminated individuals that were only found at a single ringing location in Africa (i.e. retraps at the original site of ringing). We also eliminated multiple records from any one site so that a single individual had no more than two records; all of the additional records were from the same ringing locations as one of the two records that were used, so we are confident not to have eliminated different breeding/non-breeding locations across years. Following this data filtering, our final sample size was of 125 individuals ringed in Europe and detected in Africa (or vice versa) or detected in two different locations within Africa between 1938 and 2019. EURING Data Bank mainly consisted of ringing recoveries. We are aware that there are biases regarding ringing effort across Europe and especially within Africa that may influence our interpretation.

### Statistical analyses

We averaged the latitude and longitude for each identified stationary location (stopover, breeding and wintering grounds) and calculated the duration as the number of days birds remained at the location. We used the most direct route when connecting locations, underestimating total distance travelled. Active migration refers to days taken to move from one stationary site to the next. Migration rate was calculated as the total distance covered (km) divided by the total time (days) of active migration. Minimum convex polygon areas, taking into account the curvature of the Earth, were calculated by identifying and connecting the outermost points [40] of breeding and first wintering sites separately from the five birds for whom we have information regarding their full annual cycle using the *alphahull* [41] and *geosphere* [42] R packages.

Paired *t*-tests were carried out to explore whether the duration of the first stopovers varied from the duration of subsequent stopovers. General linear models (GLMs) were used to compare the duration of stopovers according to their geographic location. Classifications according to geographical location corresponded mainly to pre- and post- barrier crossing when individuals are preparing and/or recuperating from harsh conditions and are as follows: in spring, (1) pre-Sahara/Sahel, corresponding to stopovers occurring between APLORI and before the Sahara Desert, (2) pre-Mediterranean, stopovers located in northern Africa, and (3) Europe, stopovers in Europe prior to arriving at breeding grounds. In autumn, (1) Europe, stopovers in Europe after leaving breeding grounds, (2) pre-Mediterranean, stopovers in southern Europe, prior to crossing the Mediterranean Sea), and (3) post-Mediterranean, stopovers in North Africa. Similarly, GLMs were performed to explore the relationship between the distance between APLORI and breeding grounds and the number of stopovers, duration of spring migration, departure date, and mean duration of stopovers.

Paired *t*-tests were carried out to explore whether stationary days, days in active migration, distance travelled (km), longest stationary period (days) and the number of stopovers varied between spring and autumn migration. GLMs were also used to compare arrival and departure dates with the duration of both migrations and the correlation between arrival and departure dates. Additionally, a Generalised Linear Mixed Model (GLMM) with individuals as a random effect was performed to analyse whether the total duration of migration from departure to arrival, as well as the start date of migrations (where 1 January = day 1 and excludes the first fattening period prior to departure), was different between spring and autumn migration (total migration duration ~ start date + season + (1|individual)) using the *lmer* function of the *lme4* package [43]. Throughout this manuscript, we do not consider fuelling periods before spring and autumn migration (but see Discussion). The final model did not include the interaction of date*season because it was not statistically significant. All significance thresholds were set at .05.

## Results

### Migratory routes

**Fig 1:**
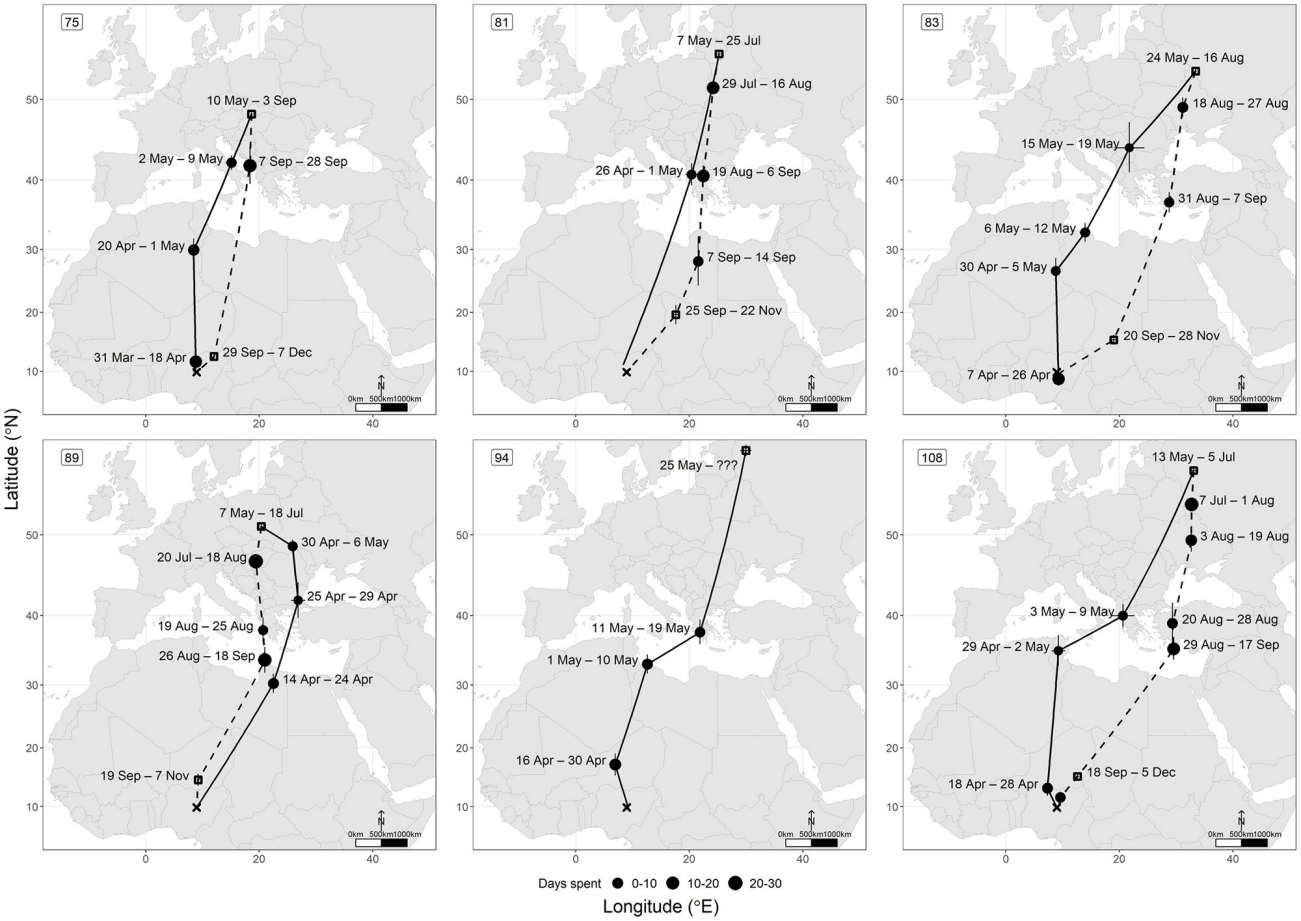
Estimated individual migratory routes. Stopovers are marked as circles and their size represents duration (days) spent at them. Squares represent breeding sites in Europe and first wintering sites in Africa. Sites are shown with standard errors plotted as black asymmetrical crosses. APLORI, in central Nigeria, is marked with a cross. Continuous lines represent spring migration and dashed lines autumn migration. We used the most direct route when connecting locations, so these may not represent exact routes. Numbers in the upper left corners reflect individual ID.

**Fig 2:**
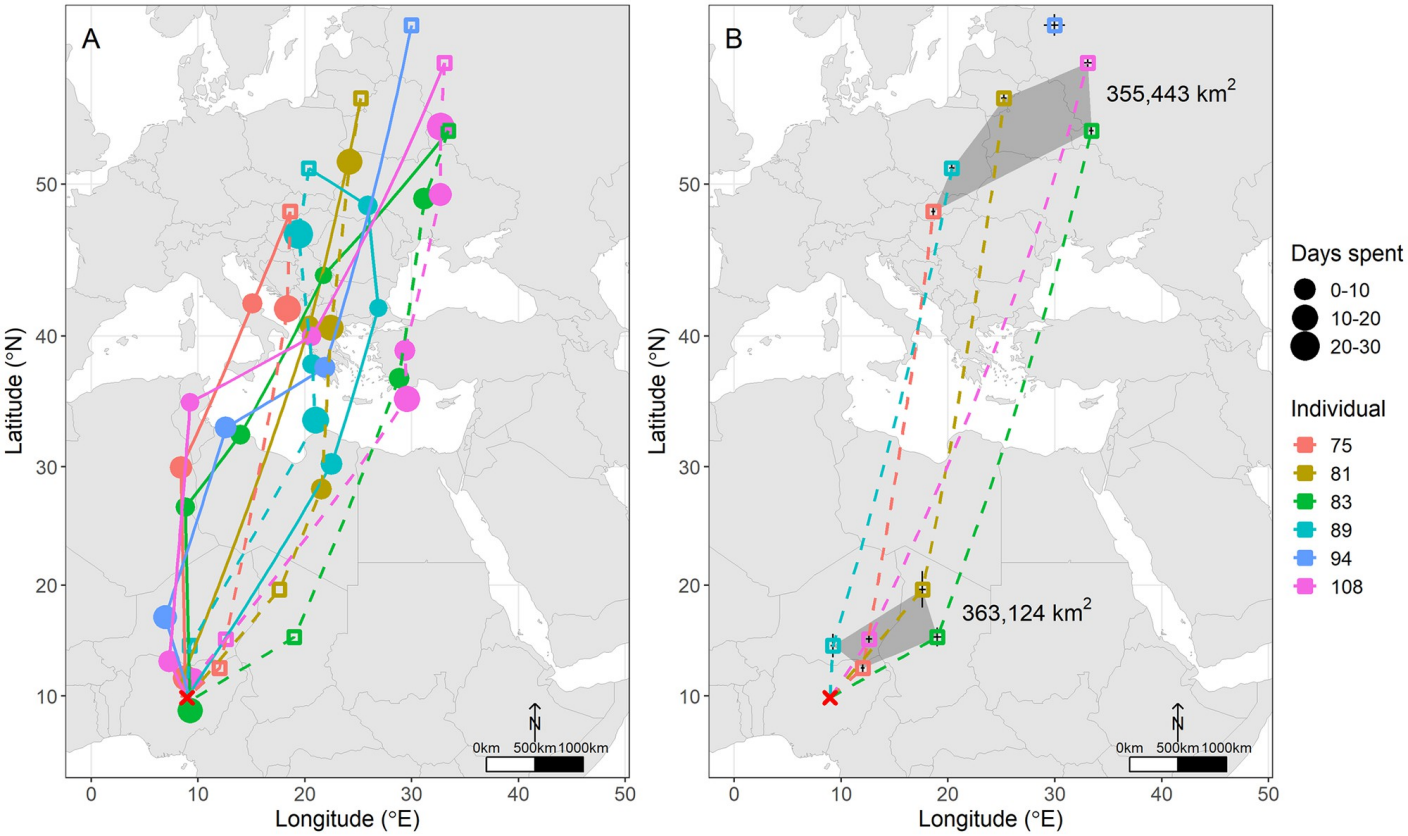
Migratory routes of Common Whitethroats wintering in Nigeria (A) and area utilised during the breeding season and at first wintering grounds (B). Map A shows the migratory routes of Whitethroats wintering in Nigeria. The size of the circle represents the time spent at stopover sites. Lines represent the most direct route from one site to the next during spring (continuous line) and autumn (dashed line) migration. Squares represent breeding grounds (in Europe) and first wintering grounds (in Africa). Map B shows the area utilised during the breeding season and at first wintering grounds. Grey convex polygons represent the connection of outer locations. Lines connect breeding sites with respective first wintering sites and first wintering sites with APLORI (represented with a red cross). Note that because there was no data regarding the first wintering site of individual 094, this individual was excluded from both polygons and area estimates. Sites are shown with standard errors plotted as black asymmetrical crosses. Each colour is a different individual.

Whitethroats left Nigeria between 30 March and 25 April and spent, on average, 32 days migrating (range = 12–48 days) until reaching their breeding grounds. Four out of six individuals flew northwardly to Algeria, Libya, or Tunisia and had a stopover site before crossing the Mediterranean Sea (Fig 1, S2 Table). The remaining two individuals flew in a north-easterly direction, where one stopped in Libya, and another flew directly to Albania (Fig 1). All individuals utilised at least one stopover site in southern Europe before reaching their final breeding grounds. All six tracked individuals arrived at their breeding grounds between 7 May and 25 May. Breeding grounds were located across eastern Europe, between Slovakia and eastern Russia (between 48°–58°N and 18°–33°E), on average, 729 km from each other, covering an area of 425,300 km^2^ (~4.2% of Europe’s area) where they remained for, on average, 81 days (range = 53–116 days) (Fig 2). The mean distance between breeding grounds and the non-breeding site at APLORI was 5,151 km (range = 4,377–5,713 km). Individuals departed breeding sites between 5 July and 3 September. They took, on average, 52 days (range = 26–75 days) to reach their first wintering site in the Sahel region. Four individuals returned following a more easterly route than their spring migration. Of those individuals, two bred in Russia—the most easterly breeders—and returned via the Black Sea in a more pronounced south-westerly direction, stopping in Turkey before crossing the Mediterranean Sea. The other two flew southwards and stopped before the Mediterranean Sea. The remaining individual returned in an anticlockwise direction via Greece. Crossing the Sahara coincides with the autumn equinox which makes our estimates of latitude and location of stopover sites during this period unreliable.

**Fig 3:**
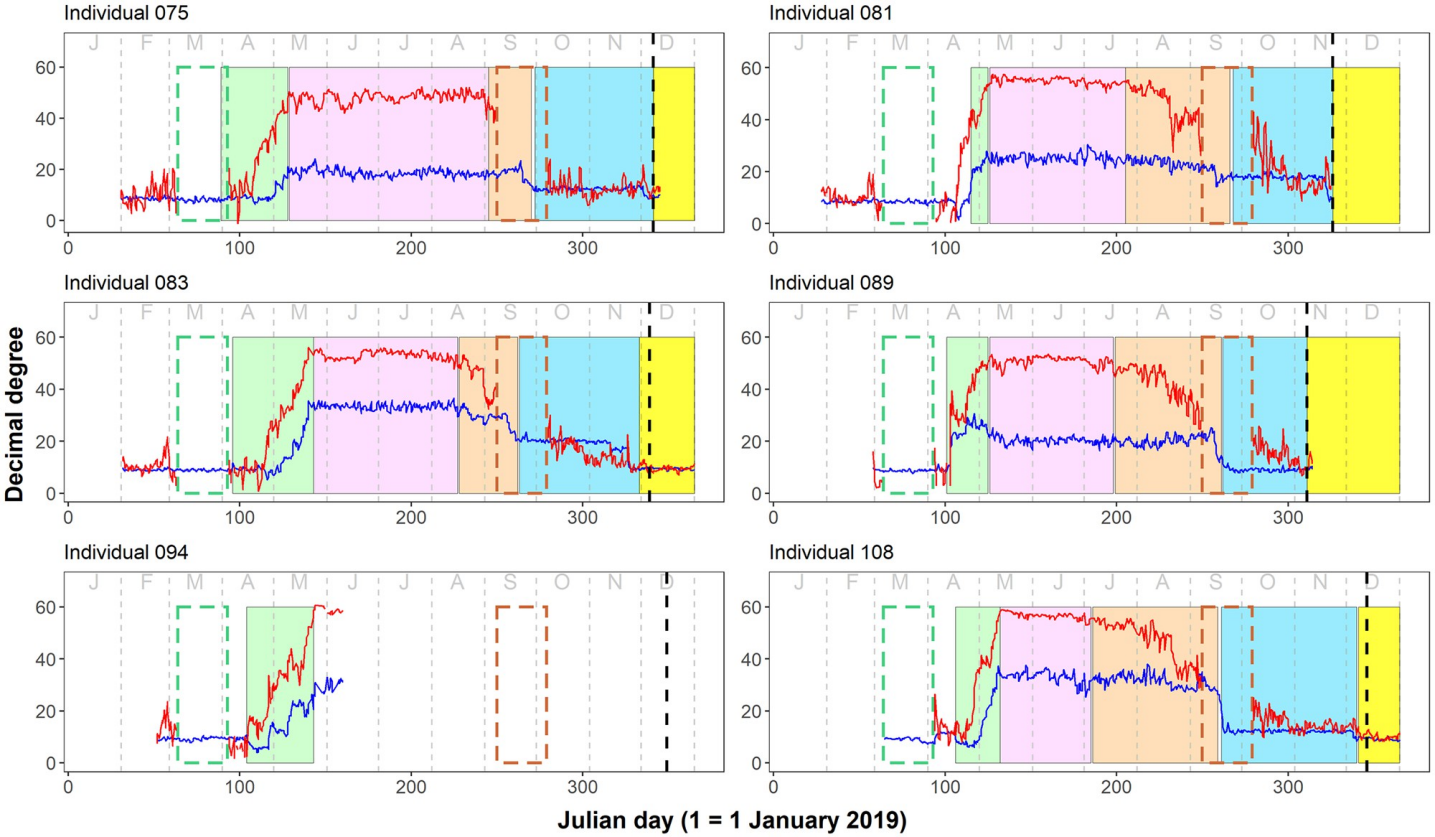
Migration phenology. Duration of spring migration (green square), breeding season (pink square) and autumn migration (orange square), and time spent at the first wintering site (blue square), and APLORI (yellow square; note that it ends on 31 December 2019 and does not reflect when individuals left the area). Annual latitudes (red line) and longitudes (blue line) of individuals. Hollow squares represent ± 14 days of equinoxes (green = spring [20 March], orange = autumn [23 September]). The black dotted line represents the day that the individual was first detected in APLORI.

Estimated individual migratory routes and areas utilised are shown in Figs 1 and 2. All five individuals utilised a first wintering site before arriving at APLORI. Individuals arrived at these sites between 18 and 29 September and remained for, on average, 65 days (range = 49–79 days). Sites were in the Sahel region, in Niger, Chad and northern Nigeria (between 12°–20°N and 9°–19°E). These were, on average, 686 km from each other, covering an area of 363,100 km^2^. The mean distance between breeding grounds and first wintering grounds was 4,300 km (range = 3,978–4,939 km) while the mean direct distance between first wintering grounds and APLORI was 875 km (range = 450–1,431 km). When considering only the five birds for whom we have information regarding their full annual cycle, we found that the area utilised during this period was similar to the area covered during the breeding season (355,400 km^2^). All individuals potentially flew directly to APLORI, the final and most important wintering site, between 7 November and 12 December and remained there until at least 10 February (Fig 3, S2 Table).

**Fig 4:**
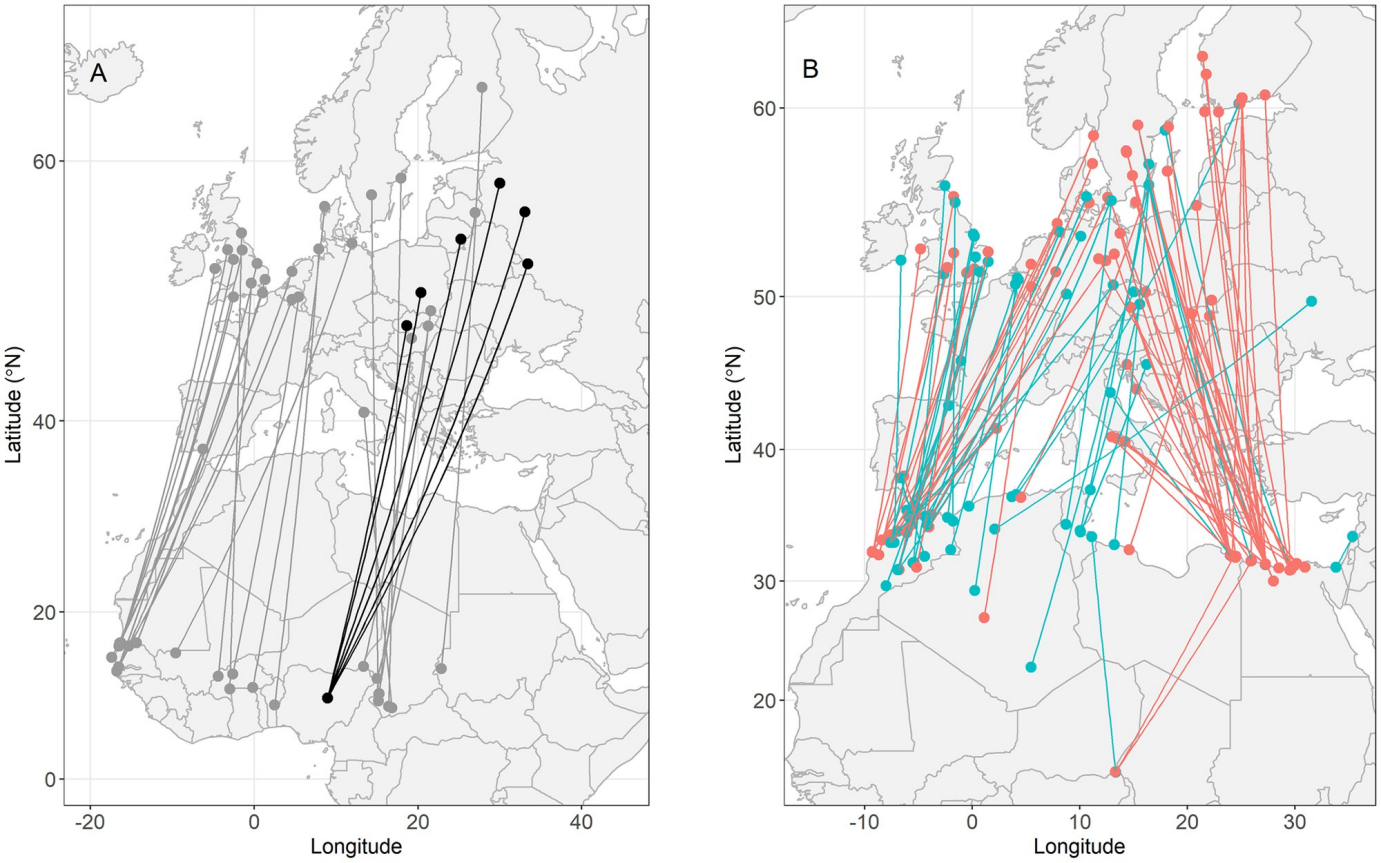
Common Whitethroats detected in both Europe and Africa. Dots represent sites where individuals were ringed and/or recaptured and lines are the most direct route between them and do not reflect migratory routes. Map A) shows 31 individuals detected in Europe and Sub-Saharan Africa. Gray dots and lines are data collected from EURING (*n* = 25) and black dots and lines represent information collected in this study through geolocators (*n* = 6). Note that for the EURING data we cannot be certain whether sites in Sub-Saharan Africa represent final wintering locations or whether European sites are breeding sites. Map B) shows information from 96 individuals that were detected in Europe and North Africa collected through EURING. Colours represent the seasonal migration when individuals were detected in North Africa, red = autumn migration (August–December) and blue = spring migration (January–July).

EURING ringing recoveries from potential breeding and non-breeding grounds also suggest a somewhat high migratory spread, as individuals from Sub-Saharan West and Central Africa bred across Europe (Fig 4A); individuals wintering at more western sites, however, breed in western-central Europe, e.g. individuals from Senegal, The Gambia, and Burkina Faso were detected in the UK, Belgium and Denmark, while individuals from central Africa, e.g. Nigeria, Chad, and Sudan, were detected further east, in northern, central, and eastern Europe, e.g. Sweden, Slovakia, Latvia, and Finland. Additionally, many individuals were detected in North Africa during spring and autumn migrations (Fig 4B). A high density of individuals from more western breeding populations seem to cross via the Strait of Gibraltar during both migrations. More eastern breeding populations, however, seem more likely to shift migratory routes depending on the season, crossing through the middle of the Mediterranean Sea in spring and favouring crossing via Egypt during autumn.

### Migratory behaviour

**Fig 5:**
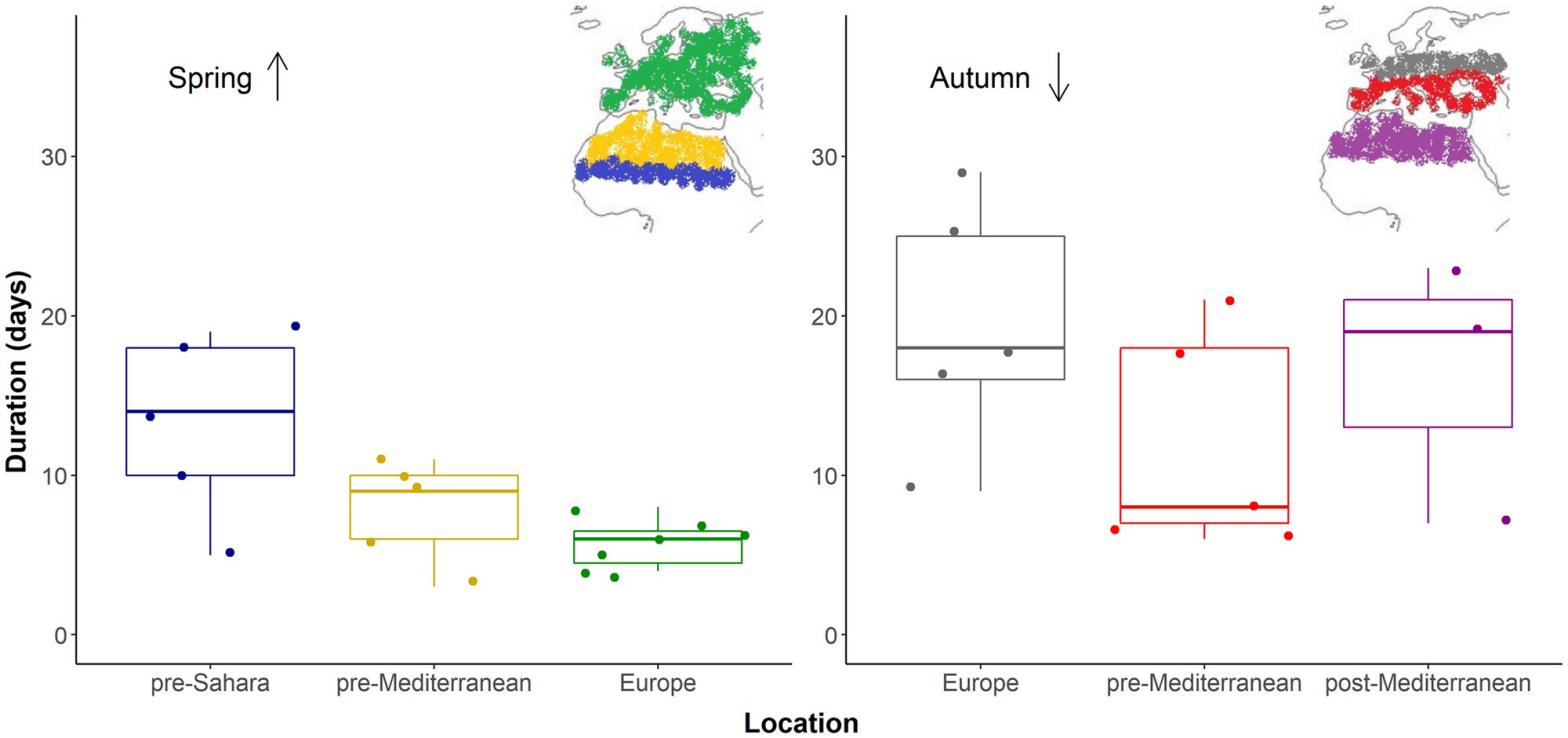
Days spent at stopovers located in different geographic locations during spring and autumn migration. In spring, blue/bottom = pre-Sahara (stopovers before the Sahara Desert), yellow/middle = pre-Mediterranean (stopovers in North Africa) and green/top = Europe (*stopovers in Europe before arriving at breeding grounds)*. In autumn, gray/top = Europe (*stopovers in Europe after leaving breeding grounds)*, red/middle = pre-Mediterranean *(stopovers in southern Europe*, *before crossing the Mediterranean Sea)* and purple/bottom = post-Mediterranean (stopovers in North Africa). The direction of arrows represents the direction of migration.

**Fig 6:**
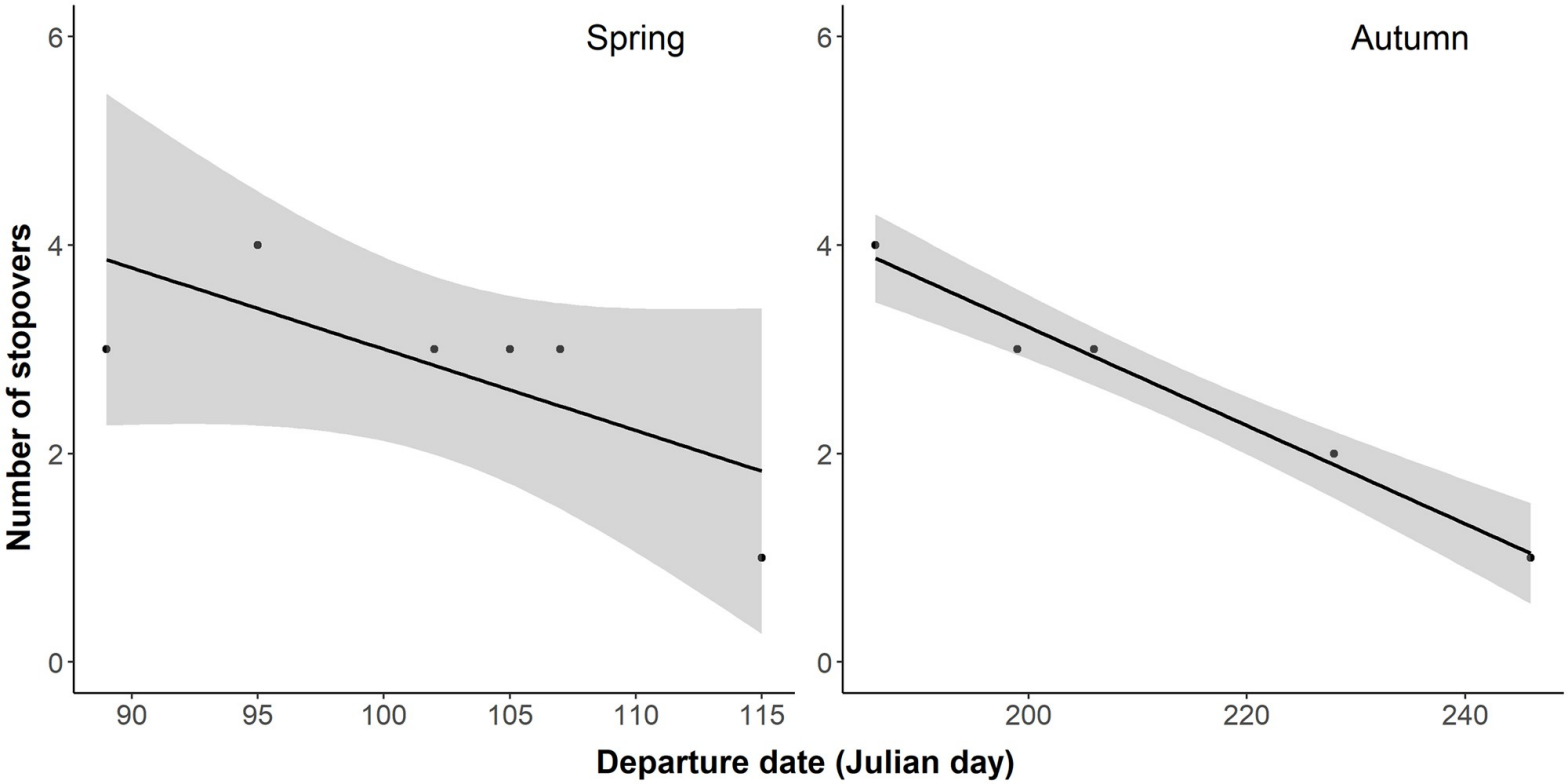
The number of stopovers in relation to spring and autumn departure dates. The lines show the estimates for the effect of departure dates on number of stopovers, and gray areas reflect the 95% confidence intervals for the estimates.

All individuals carried out stopovers during active migration. The number of stopovers was similar during spring and autumn migration (mean spring = 2.8; mean autumn = 2.6). In spring, the duration of the first stopover was significantly longer compared to the rest (paired *t*-test: *t* = 4.6, df = 4, *p* = .01). Similar results were obtained in autumn (paired *t*-test: *t* = 3, df = 3, *p* = .06), though were not significant at *p* < .05. When comparing the duration of stopovers according to their geographic location in spring, birds undertook longer stopovers at the pre-Sahara/Sahel locations compared to sites in the pre-Mediterranean and Europe (*F*_(2,14)_ = 6.1, *p* = .01) (Fig 5). In autumn, however, the duration spent at stopovers did not vary according to geographic location (*F*_(2,10)_ = 1.2, *p* = .34) (Fig 5). Statistical power is limited by sample size but the number of stopovers during autumn migration was strongly related to departure date from breeding grounds (*F*_(1,3)_ = 149.8, *p* = .001): individuals that departed earlier from breeding grounds had more stopovers than later-departing individuals (Fig 6). A similar trend was found during spring migration (F_(1,4)_ = 4.5, *p* = .1). Whitethroats covered > 4,000 km, and actively migrated for an average of 9.3 days (± 5.2 SD), leading to an estimated average migration rate of 690 km/day (± 277 SD; distance/flying duration).

Statistical power is again limited in all cases but the distance travelled between APLORI and the breeding grounds during spring did not appear to correlate with the number of stopovers (F_(1,4)_ = 0.6, *p* = .50), duration of spring migration (F_(1,4)_ = 0.03, *p* = .87), departure date from APLORI (F_(1,4)_ = 0.7, *p* = .45) or mean duration of stopovers (F_(1,4)_ = 0.4, *p* = .57). Similar results were obtained during autumn migration: the distance between breeding grounds and first wintering sites did not correlate with the number of stopovers (F_(1,3)_ = 2.8, *p* = .19), duration of autumn migration (F_(1,3)_ = 1.6, *p* = .30), departure dates from breeding grounds (F_(1,3)_ = 2.3, *p* = .23) and mean duration of stopovers (F_(1,3)_ = 0.01, *p* = .91).

### Differences between spring and autumn migration

Paired *t*-tests reported no statistical differences in the mean number of stationary days, days of active migration, longest staging period, or number of stopover sites between spring and autumn migration (Table 1). However, distance travelled was significantly greater and speed was faster during spring migration (Table 1). Arrival dates did not correlate with departure dates in either spring (Pearson correlation coefficient (*r*) = -0.2, *t* = -0.4, df = 4, *p* = .68) or autumn (Pearson correlation coefficient (*r*) = 0.7, *t* = 1.9, df = 3, *p* = .15). Autumn migration was overall longer in duration than spring migration (Table 2). Similarly, there was a strong negative linear effect between the timing of departure from breeding or final wintering grounds and duration of migration: birds that migrated earlier undertook longer migrations (Table 2).

**Table 1:** Spring and autumn migration details. Results from paired *t*-tests. All data from individual 094 was excluded. The time spent fattening at APLORI and breeding grounds during spring and autumn migration, respectively, are not considered in estimates.

	Spring		Autumn		*Statistical results*
	*Mean*	*SD*	*Mean*	*SD*	
Stationary (days)	22.8	12.6	41.2	22.6	*t* = 1.3, df = 4, *p* = .27
Active migration (days)	7.6	3.7	11	7.4	*t* = 1.4, df = 4, *p* = .22
Longest staging period (days)	12.4	5.9	20.4	7.6	*t* = 1.5, df = 4, *p* = .2
Distance (km)	5,524	619	4,415	487	*t* = -5.3, df = 4, *p* = .006*
Migration rate (km/day)	806	268	574	324	*t* = -2.9, df = 4, *p* = .04*
Number of stopovers	2.8	1	2.6	1.1	*t* = -0.3, df = 4, *p* = .82

* statistically significant values *p* < .05.

**Table 2:** Results from a generalised linear mixed model regarding total migration duration. Formula: total migration duration from departure to arrival ~ start date + season + (1|individual).

Variable	Estimate	Std. Error	df	*t*-value	*p*-value
(Intercept)	124.5	14.2	8	8.2	< .001
Start of migration	-0.9	0.1	8	-6.2	< .001
Season (spring/autumn)	120.3	15.6	8	7.2	< .001

## Discussion

We describe, for the first time, the complete annual cycle of a small sample of Whitethroats. We have shown that individuals wintering in central Nigeria breed across eastern Europe, indicating a somewhat high migratory spread defined by a north-eastern flight pattern. Data also show that individuals migrate primarily using intermittent migration, stopping for longer periods at a first stopover location after leaving final wintering and breeding grounds, especially during spring migration prior to crossing the Sahara Desert. If under severe time constraints, however, individuals can successfully undergo a flight without making stopovers. During spring migration individuals travelled longer distances at faster rates making it overall shorter than autumn migration. There was clear evidence that Whitethroats undertake loop migration, mainly in a clockwise direction, and visit multiple wintering sites: first in the Sahel region, followed by a more south-westerly site, APLORI, where they remain until spring migration.

### Migratory spread

The location of Whitethroats’ breeding grounds revealed by geolocators is in line with our prediction and with previous knowledge gained through years of ring recoveries: Nigerian individuals fly in a north-easterly/south-westerly direction during migration, and breed in a large area throughout central, northern, and eastern Europe [27, 29]. These results are also consistent with EURING long-term ringing dataset (Fig 4A) and with the recapture of a Nigerian-ringed individual in Poland in 2000.

Individuals from a small six km^2^ wintering site at APLORI bred between Slovakia and Russia and occupied a first wintering site between Nigeria and Chad, in a roughly similarly sized area (~360,000 km^2^). These results suggest that Whitethroats have a high degree of migratory spread consistent with the general pattern from other studies [44], though Afro-Palearctic migrants show different degrees of migratory spread, ranging from extremely high spread, e.g. Whinchats *Saxicola rubetra* [21] and Great Reed Warblers *Acrocephalus arundinaceus* [45], to moderate spread, e.g. Great Reed Warblers [17], and low spread, e.g. Common Nightingales *Luscinia megarhynchos* [46] and Pied Flycatchers *Ficedula hypoleuca* [47].

The degree of migratory spread has important effects on the ecology and conservation of migratory birds and will affect a species’ response to changing environmental pressures [48]. On one hand, a population that relies on few non-breeding sites (low spread) is expected to have stronger local adaptations (e.g. food, predators, climate) and a higher vulnerability if there were to be any changes at those particular sites [49]. On the other hand, populations showing a high spread should have generalist-associated traits and greater potential to track shifting habitats while being more resilient to these changes [5, 48]. However, high spread implies higher probabilities of inter-population mixing at the non-breeding grounds, so changes occurring there would have “small” effects on many breeding populations [44].

### Migratory behaviour

Whitethroats have been suggested, on the one hand, to undergo an intermittent migration flying from one stopover to the next [26, 27], and on the other, to undergo a non-stop flight crossing the Sahara Desert and Mediterranean Sea in a single journey [28]. Our results suggest that while Whitethroats are able to carry out both migratory strategies, they undertake an intermittent flight to restore their body condition prior to and after crossing major barriers [50]. All individuals stopped in North Africa during spring migration except for one individual that probably crossed the Sahara and the Mediterranean Sea in a single non-stop flight (i.e. had no stopovers greater than three days). The exceptional bird was the last individual to leave APLORI on 25 April and, thus was a late, faster travelling bird under more time constraint. Non-stop migration was previously observed in a high proportion of Whinchats tracked from APLORI [51]. During the autumn equinox, there is much more uncertainty regarding barrier crossing due to the difficulties of tracking during the equinox, but evidence suggests that Whitethroats fly to a first wintering site several hundred kilometres north of the final wintering site at APLORI, potentially after undertaking a single non-stop flight from southern Europe. This could be possible as, during the end of summer, resources are likely to still be available throughout the route and long stopovers may not be necessary.

Our results also emphasize the importance of first stopover sites after leaving the final winter and breeding grounds. In spring especially, we found that individuals left their wintering territories at the end of the dry season to build fat reserves in the Sahel close to the southern limit of the Sahara Desert. This region, therefore, represents an important site during spring migration and further highlights the importance of the presence and availability of *Salvadora persica* berries, which are an important resource for pre-migratory fattening for the species [52].

### Seasonal migration differences

Total migration duration among birds depends strongly on environmental conditions and fuel deposition rates [53], and selection pressures differ according to seasonality [12]. The total duration, from departure to arrival, of spring migration, despite it involving longer distances, was much quicker than autumn migration, lasting between 12 and 51 days and between 26 and 75 days, respectively, but we did not find any strong differences between spring and autumn migration regarding days spent stationary, days in active migration, longest staging period, and number of stopovers. These results support the optimal migration theory where timing of migration is more critical in spring than in autumn: birds exhibit overall quicker migrations during spring [54, 55] to ensure timely arrival at breeding grounds to occupy and establish better territories and find higher-quality mates [56, 57], while arriving later at the non-breeding grounds has fewer fitness consequences [58]. Individuals also need to ensure that they do not arrive *too* early when weather is unfavourable and peak resource availability is yet to occur, but must still attempt to arrive earlier than others to compete for territories—especially when arriving at breeding grounds [57].

We note that migration is, to a large extent, feeding, so feeding before departure for spring and autumn is an important component of total migration duration [59] that was not considered during this study. We can, however, use the theoretically predicted 1:7 relationship between flight and stopover times proposed for small birds [60]. From our study, the average number of days spent on active migration was 7.6 in spring and 11 in autumn, and the estimated stationary days were 22.8 and 41.2 days, respectively (Table 1). Assuming that the fuel deposition rate is constant throughout migration, an individual may therefore be predicted to spend a total of 53.2 and 77 days fuelling in spring and autumn, respectively. Thus, the duration of the first fuelling period should, in theory, be 30 days in spring and 36 days in autumn and total migration duration should be 62.5 and 89.2 days, respectively.

### Loop migration

The use of different migratory routes during autumn and spring migration is a common strategy for several Afro-Palearctic migrants. Loop migration patterns (clockwise when birds take a more easterly route in autumn, anticlockwise when reversed) likely emerge from a combination of adaptations to dominant wind systems, ecological barriers, spatiotemporal distribution of resources and suitable habitats and historical aspects of the species’ distribution [15]. Loop migrations reduce overall migration duration, mitigate the risk of running out of fuel and increase migration speed, despite increasing overall distance travelled. In Mauritania, a higher number of Whitethroats were trapped during spring than autumn, suggesting a different migratory route between seasons [61], and supporting our findings.

The direction of the loop seems to vary substantially according to the location, population and species and might be related to a combination of wind direction and food availability [17, 45, 54]. A few species exhibit a clockwise direction, e.g. Turtle Doves *Streptopelia turtur* [50], but most seem to show an anticlockwise direction, e.g. Barn Swallows *Hirundo rustica* [15] and Whinchats [16], or both, e.g. Great Reed Warblers [17].

In this study, we found intraspecific variation, as four of our tracked individuals showed a clockwise loop migration and one an anticlockwise direction. The odd one out did not show any obvious difference in its breeding location. If winds were the strongest predictor in the direction of the loop, we would expect all individuals or similar species that are in roughly the same areas to have the same loop direction. Our results suggest that there are other selective pressures determining loop direction, but larger sample sizes are needed to test this.

The EURING data suggests that loop migration may be more pronounced in eastern populations and even non-existent in western populations (Fig 4B), at least when migrating between North Africa and Europe. This could be explained by barriers posed by the Mediterranean Sea and the Strait of Gibraltar. Because large water bodies represent major obstacles for migratory birds, western populations may opt to migrate on land for as long as possible and avoid the Mediterranean crossing altogether. This suggests that the advantages of loop migration may not exceed the disadvantages of crossing bodies of water. If true, then loop migration may be highly population-specific, depending on geographical traits and landmass, and age-dependent if first years have less knowledge and assume more risks than adults [62]. This, however, is merely speculative and further research is required, especially within Africa.

### Migration rate

Whitethroats had an estimated average migration rate of 690 km/day, considerably higher than reports from other Whitethroat studies (< 250 km/day) [56, 63]. Assuming birds fly continuously through the day and night, the average minimum flight speed is 29 km/h. Because of low geolocator resolution, this method does not allow the detection of short stops (< three days) and assumes linear flight paths, so we expect this speed to be an underestimation of real average flight speed. Typical flight speeds of migrating passerines the size of Whitethroats has been measured in the field as an average of 45 km/h, potentially much higher with wind assistance [64, 65]. Whitethroats could therefore be dedicating effort to flying overnight and resting and/or refuelling during much of the day.

### Multiple site use

There is a continuum of residency and only a subjective distinction between when a long stopover site becomes a stationary period, but long stationary periods are perhaps best defined as locations where individuals spend more time than needed just for refuelling. The use of multiple wintering sites could be part of a strategy in which birds temporarily suspend migration to optimise resource use during the non-breeding season, based on predictability of food sources in the region [66], and is strongly related to environmental conditions [67, 68]. All five individuals tracked throughout their complete annual cycle utilised a first wintering site prior to arriving at APLORI (~900 km away), where they remained for an average of two months (September–November). One individual spent more time at this first site than at the breeding grounds. Due to the presence of Whitethroats throughout the complete non-breeding season at APLORI (September–April), we believe APLORI to be an important non-breeding ground for Whitethroats, acting as a second site for some birds, and a first site for others.

The use of several wintering sites seems to be common for long-distance migrants. Many other species have been found to have up to two (Red-backed Shrikes *Lanius collurio* [54] and Whinchats [16]), three (Great Reed Warblers [17, 45]), and four sites (Purple Martins *Progne subis* [69]). Whitethroats arrive at the Sahel savannah, in central-eastern Africa at the end of the summer rains. During this period, habitats are green and productive and insect abundance is high, representing favourable foraging conditions [19, 54]. As time passes, habitats dry and conditions become harsh [11] causing birds to move to their final wintering grounds between November and December where rainfall ends later and conditions remain sufficient to survive the season.

Seasonal rainfall patterns are the main driving force for the distribution and movement of migratory birds [14]. In South America, Veeries *Catharus fuscescens* are thought to relocate from one wintering site to another due to predictable seasonal flooding, so they select lowland forests as an initial wintering site and relocate to higher elevations or unflooded regions as the rainy season progresses [67]. APLORI may provide a more secure environment for Whitethroats to spend the harshest non-breeding periods because they remain for over four months. Whinchats—another common Afro-Palearctic migrant in APLORI—tagged in the UK also had second non-breeding sites located 400 km to the west of the first [16].

### Conservation implications

The future of a species depends not only on its ability to adapt but on efficient conservation strategies at both the breeding and non-breeding grounds that will buffer the impact of future climatic and anthropogenic changes [70]. We identified the Sahel region as an important refuelling site during spring migration and as a first wintering site in autumn. The condition of the Sahel is crucial for the species [71] and this is regulated by rainfall patterns [72]. A population crash observed in the 1960s showed how susceptible the species can be to major climate changes in the region [73], but also demonstrated the species’ ability to recuperate and adapt [74]. Their potential generalist traits, their use of a wide variety of resources throughout the year and their flexibility to use different migratory strategies might make this species resilient to certain changes. We believe that because Whitethroats have a relatively high migratory spread, changes in the central sub-Saharan African non-breeding grounds will have severe effects on a subset of individuals of specific European breeding populations (e.g. Polish, Belarussian, Lithuanian, western Russian), but due to Whitethroats’ large distribution, the most western and eastern populations may not be as severely affected [17]. However, the effects these changes will have on the species will greatly depend on the degree of change and at what spatial scale changes occur.

Based on migration tracks, the average annual Whitethroat time allocations for non-breeding sites, spring migration, breeding, and autumn migration are 55%, 9%, 22% and 14%, respectively. Africa, where individuals spend < 65% of the year, is a continent of rapid social and economic change that will have a strong impact on their resources and natural habitats. Strong land-use changes at these sites will have a diffuse impact on many Whitethroat breeding populations, so for alarming impacts to occur at a species level, these changes would need to occur at a very large scale. If this ensues, protection efforts will need to include larger geographic areas and consequently, greater logistic challenges would arise [6]. Further full annual cycle studies of Afro-Palearctic migrants are needed to identify where species are more susceptible so that conservation efforts can be directed accordingly [3, 75], either by protecting one large area or focusing on several small ones. Small spatial movements throughout the annual cycle will become clearer when non-archival tags such as satellite transmitters and/or GPS devices are sufficiently lightweight to be fitted on small birds throughout the year.

## Supporting information

S1 Table Geolocator data. Mean weight and SE of each geolocator model with and without a harness, and the number of control birds and individuals deployed with each geolocator model according to age and sex (F = female, M = male, U = unknown). The numbers in parenthesis indicate the number of individuals that were recovered and/or seen the following year. Photographs below show the different geolocator models fitted on individuals using an elastic leg-loop harness following Rappole and Tipton (1991).
(PDF)

S2 Table Details regarding spring and autumn migration, breeding season and first wintering site. (XLSX)
