# Supplementary material for: Migratory routes, breeding locations and multiple non-breeding sites of Common Whitethroats Curruca communis revealed by geolocators
Source: PLoS One. 2022 Sep 1;17(9):e0274017. doi: 10.1371/journal.pone.0274017 (PMC9436143; doi:10.1371/journal.pone.0274017)
Supplement: S1 Table — Mean weight and SE of each geolocator model with and without a harness, and the number of control birds and individuals deployed with each geolocator model according to age and sex (F = female, M = male, U = unknown). The numbers in parenthesis indicate the number of individuals that were recovered and/or seen the following year. Photographs below show the different geolocator models fitted on individuals using an elastic leg-loop harness following Rappole and Tipton (1991). (PDF) [file pone.0274017.s001.pdf]

Table S1. Geolocator data.
Mean weight and SE of each geolocator model with and without harness, and the number of control birds and individuals deployed with each geolocator model according to age and sex (F = female, M = male, U = unknown). The numbers in parenthesis indicate the number of individuals that were recovered and/or seen the following year. Photographs below show the different geolocator models fitted on individuals using an elastic leg-loop harness following Rappole and Tipton (1991).

Model	Weight without harness (g)	Weight with harness (g)	% of body weight	Adult			First-year			U age	Total
				F	M	U	F	M	U		
ML6740	0.51 ± 0.03	0.57 ± 0.03	4.0 ± 0.6	13	10	0	0	3	13	1	40 (6)
FL6B57	0.40 ± 0.01	0.45 ± 0.02	3.2 ± 0.3	1	6	0	0	0	3	0	10 (1)
FL6057	0.37 ± 0.01	0.43 ± 0.01	3.1 ± 0.4	3	1	1	1	3	1	0	10 (0)
		Geolocated birds		**17** (4)	**17** (3)	**1** (0)	**1** (0)	**6** (0)	**17** (0)	**1** (0)	**60** (7)
		Control birds		**8** (1)	**15** (3)	**6** (1)	**1** (1)	**4** (0)	**23** (3)	**3** (0)	**60** (9)

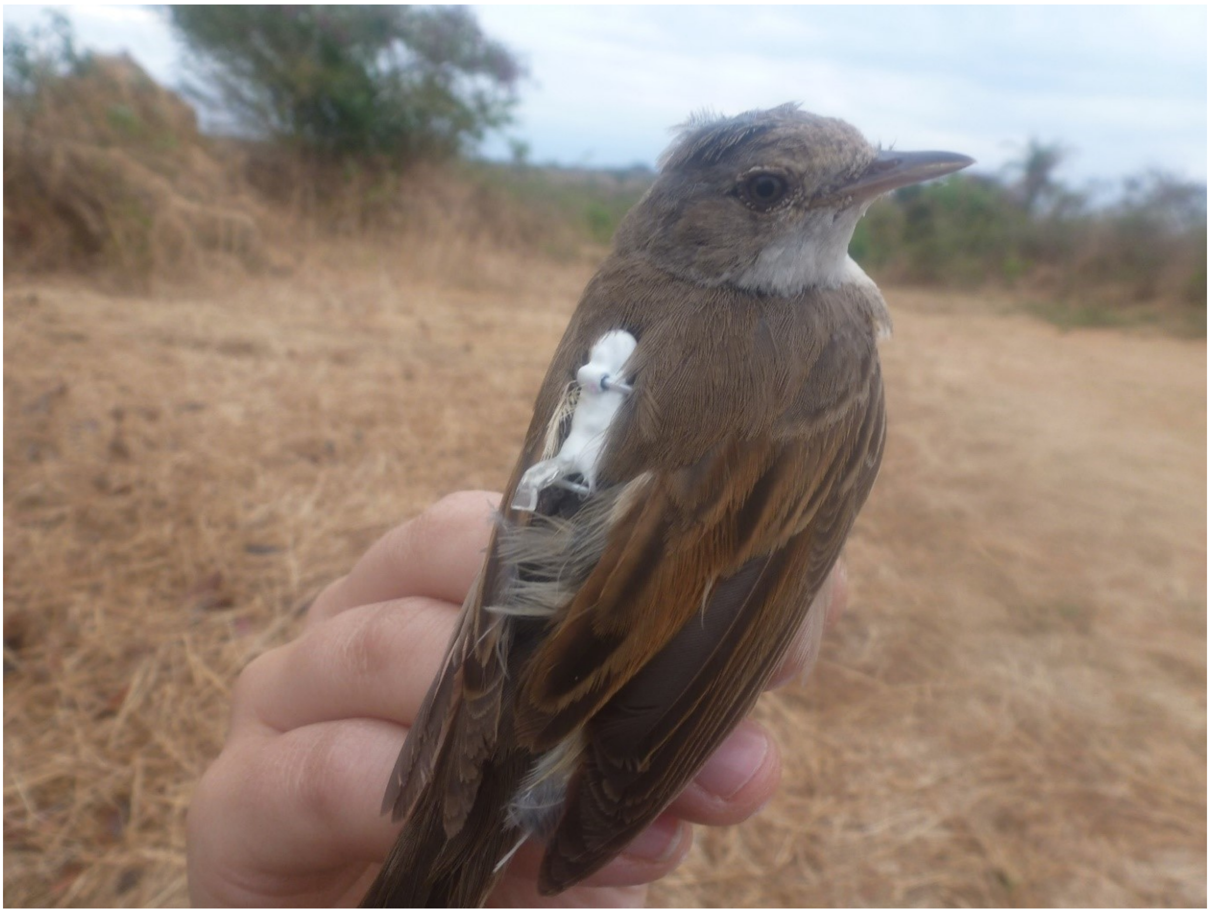

Photo 1. Example of a "ML6740" geolocator. Developed by the *British Antarctic Survey*. This model has a 5 mm light-stalk positioned at a 45° fixed angle.

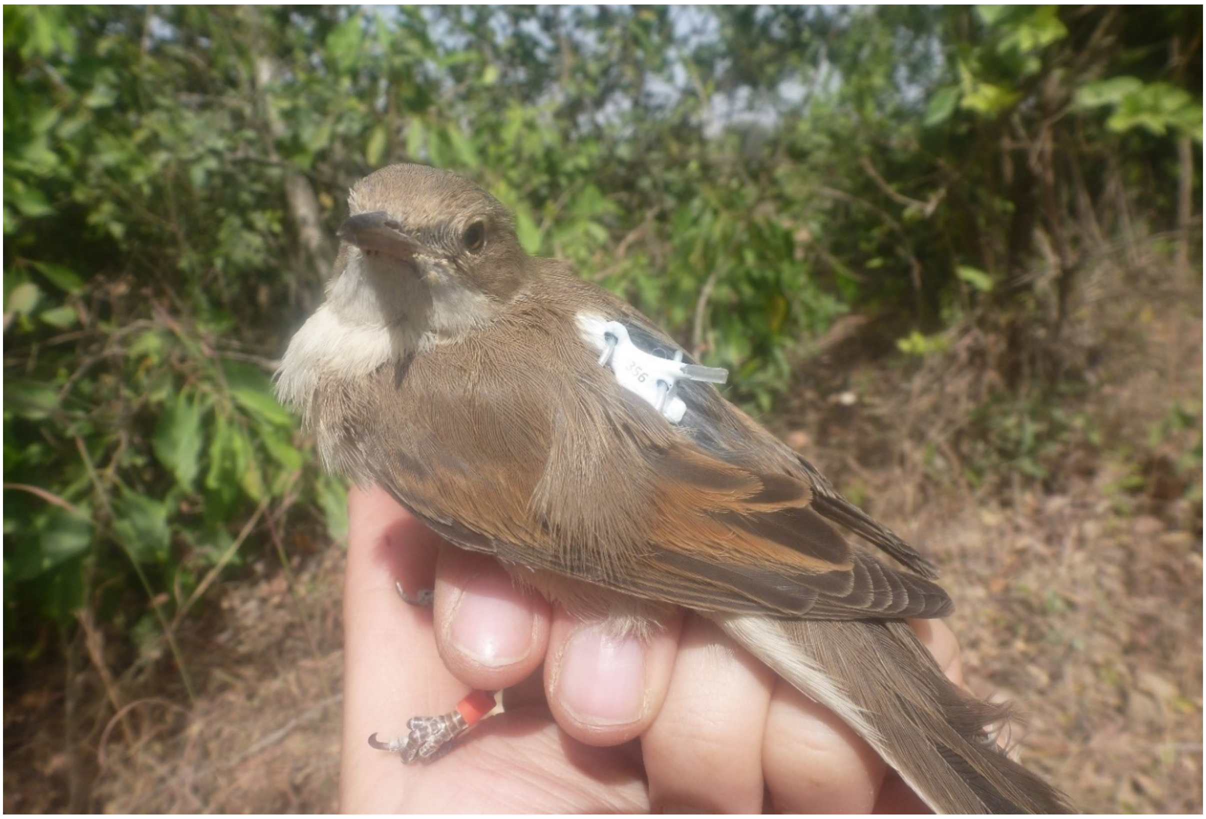

Photo 2. Example of a "FL6B57" geolocator. Developed by *Lotek/Biotrack*. This model has a 5 mm light-stalk positioned at a 45° fixed angle. Records light every 4 minutes.

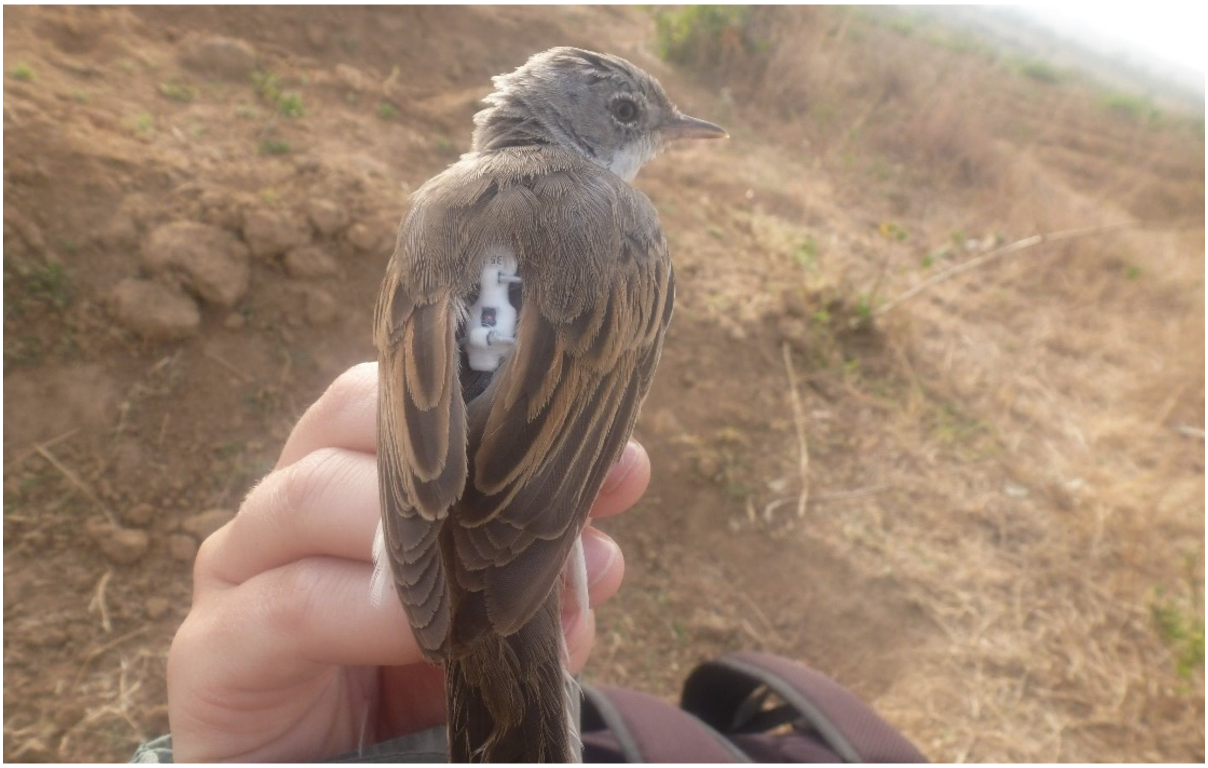

Photo 3. Example of a "FL6057" geolocator. Developed by *Lotek/Biotrack*. This model is the lightest, as it does not have a light-stalk. Records light every 4 minutes.
